# Isobologram analysis of triple therapies

**DOI:** 10.1186/1748-717X-1-39

**Published:** 2006-10-17

**Authors:** Maximilian Niyazi, Claus Belka

**Affiliations:** 1 CCC Tübingen, Department of Radiation Oncology, Hoppe-Seyler-Str. 3, 72076 Tübingen, Germany

## Abstract

New concepts in radiation oncology are based on the concept that combinations of irradiation and molecular targeted drugs can yield synergistic or at least additive effects. Up to now the combination of two treatment modalities has been tested in almost all cases. Similar to conventional anti-cancer agents, the efficacy of targeted approaches is also subject to predefined resistance mechanisms. Therefore, it seems reasonable to speculate that a combination of more than two agents will ultimately increase the therapeutic gain. No tools for a bio-mathematical evaluation of a given degree of interaction for more than two anti-neoplastic agents are currently available.

The present work introduces a new method for an evaluation of triple therapies and provides some graphical examples in order to visualize the results.

## Background

Many mathematical approaches have been described in order to determine the level of interaction of two agents. In this regard, isobologram analysis was developed and described 30 years ago and is still the most popular tool for this question [1,8]. Basically, isobologram analyses are an approach to represent zero-interaction curves of two agents. However, classical isobologram analyses are quite resource intensive and therefore a widespread use has never been adopted.

Although the combination of two agents was effective in many clinical settings, a combination of three or more treatment principles is even more realistic.

In case of radiation oncology it has been shown that the inhibition of EGF-R in combination with radiation using the C225 antibody was effective in terms of local control and survival [4]. However, cis-platinum based radiochemotherapy represents the current standard approach for advanced head and neck cancer. Currently the combination of radiation, cis-platinum and C225 is tested clinically while still lacking a complete preclinical evaluation of the combined therapy [7].

Although targeted agents are clearly effective [6], like for conventional agents the long term efficacy is hampered by specific resistance mechanisms. Therefore it seems to be likely that in the future combinations of distinct and/or interactive targeted drugs will be used in clinical settings.

The present work provides a new mathematical formalism to analyse the level of interaction of three treatment approaches based on a reduced scale data set.

### Theoretical background

Before introducing any mathematical detail, it is of crucial importance to define the terms used within this paper: The semantic definition of synergy describes an interaction that is more effective than the sum of the single effects (known by the famous holistic saying "the whole is more than the sum of its parts"). Therefore the term synergy or "supra-additivity" describes situations where the combination of agents acts more than additive [2].

The two classical definitions of additivity go back to Loewe [5] and Bliss [3]. Bliss developed the model of response additivity which is also called the criterion of Bliss independence. These definitions are not only formal thoughts but do have some practical implications [8] which are especially important in the field of radiation oncology. Response additivity means that we assume statistical independence which leads to a pure addition of the effects. In contrast, dose-additivity assumes that the agents behave like simple dilutions and act without self-interaction. In this case it has become popular to talk of zero-interactive responses.

For this purpose Berenbaum developed the following formula:

∑jdjDj=1     [1]
 MathType@MTEF@5@5@+=feaafiart1ev1aaatCvAUfKttLearuWrP9MDH5MBPbIqV92AaeXatLxBI9gBaebbnrfifHhDYfgasaacH8akY=wiFfYdH8Gipec8Eeeu0xXdbba9frFj0=OqFfea0dXdd9vqai=hGuQ8kuc9pgc9s8qqaq=dirpe0xb9q8qiLsFr0=vr0=vr0dc8meaabaqaciaacaGaaeqabaqabeGadaaakeaadaaeqbqaamaalaaabaGaeeizaq2aaSbaaSqaaiabbQgaQbqabaaakeaacqqGebardaWgaaWcbaGaeeOAaOgabeaaaaaabaGaeeOAaOgabeqdcqGHris5aOGaeyypa0JaeGymaeJaaCzcaiaaxMaacqGGBbWwcqaIXaqmcqGGDbqxaaa@3C59@

where d_i _is the actual dose (concentration) of the individual agents in a combination and *D*_*i *_is the dose (concentration) of the agents that individually would produce the same effect as the individual compounds in the combination [1].

By handling linear dose-response-curves one only gets a straight line of additivity which divides the plane into the areas "supra-additive" and "infra-additive".

As one usually considers dose-response-relationships that are non-linear, these two concepts will lead (in the case of two agents/modalities) to an envelope of additivity.

**Figure 1:**
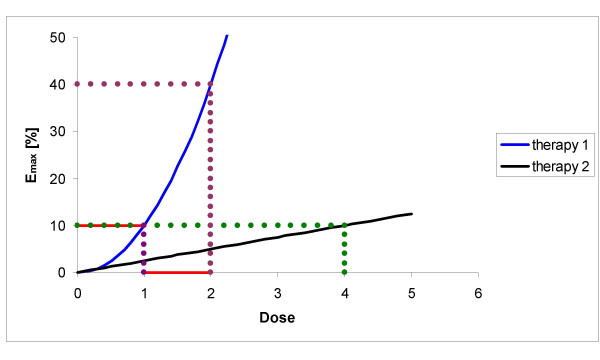
In this diagram two dose-response-relationships are plotted whereas E_max _denotes the fraction of the maximum effect. Therapy 1 is quadratic (y = 10 x^2^) and therapy 2 is linear (y = 2,5 x). One needs one dose unit of therapy 1 to obtain 10% of the maximum effect and four dose units of therapy 2 for the same effect; so a combination would yield (in the strict response additive case) 20%. In the case of Loewe-additivity one would analyse as follows: therapy 2 yields the same like one unit of therapy 1. So the effect would be the same as for two units of therapy 1, namely 40%.

The different concepts are made clear by an example (see Fig. 1):

If one assumes a quadratic dose-response relationship for one of the agents (therapy 1) and a linear relationship for a less effective drug (therapy 2) and if one furthermore needs one dose unit of therapy 1 to obtain 10% of the maximum effect (E_max_) and four dose units of therapy 2 for the same effect, a combination would yield (in the strict response additive case) 20%. In the case of Loewe-additivity this would lead to the following: therapy 2 yields the same like one unit of therapy 1. So the effect would be the same as for two units of therapy 1, namely 40%!

### Formal definitions

Now one has to focus on the effect of the single agents. The effect can e. g. be the rate of apoptosis, the amount of dead cells or something similar. The following theory can readily be modified and is completely analogous if one measures surviving fractions. In this case -ln(SF) has to be regarded as the effect where the logarithm is a contribution to the definition of the suriving fraction; this means that surviving fractions are multiplicatively connected while the natural logarithms are additive.

In order to describe the relationship mathematically, the following notation is introduced:

y_j _= f_j _(x_j_), j ∈ {1, 2, 3},

where y denotes the effect and x the dose/concentration of XRT or the drug (in the following the term "dose" is used for both concepts). The functions f_j _should be continuous and invertable (i. e. bijective). The inverse functions will be denoted by f^-1^_j_.

Let d_j _be the dose of the jth modality in the triple therapy. Therapies will be denoted as (d_1_|d_2_|d_3_); d_1 _relates to the concentration of a hypothetical agent A [μg/ml] (the units are suppressed during the calculations), d_2 _is the concentration of another agent B [nM] and d_3 _denotes the dose of (X)RT [Gy].

### Concept

First the surface of strict response additivity will be introduced. It is the surface that contains all combinations which would produce an effect that equals the effect of the triple therapy (the corresponding dose is called isoeffect-dose or in brief isodose). It is denoted by "i". The first formula may thus be written as

∑j=13fj(xj)=i     [2]
 MathType@MTEF@5@5@+=feaafiart1ev1aaatCvAUfKttLearuWrP9MDH5MBPbIqV92AaeXatLxBI9gBaebbnrfifHhDYfgasaacH8akY=wiFfYdH8Gipec8Eeeu0xXdbba9frFj0=OqFfea0dXdd9vqai=hGuQ8kuc9pgc9s8qqaq=dirpe0xb9q8qiLsFr0=vr0=vr0dc8meaabaqaciaacaGaaeqabaqabeGadaaakeaadaaeWbqaaiabbAgaMnaaBaaaleaacqqGQbGAaeqaaOWaaeWaaeaacqqG4baEdaWgaaWcbaGaeeOAaOgabeaaaOGaayjkaiaawMcaaaWcbaGaeeOAaOMaeyypa0JaeGymaedabaGaeG4mamdaniabggHiLdGccqGH9aqpcqqGPbqAcaWLjaGaaCzcaiabcUfaBjabikdaYiabc2faDbaa@41C7@

which represents a three-dimensional surface in space.

The presented semantic definitions furthermore lead to the following seven equations:

f^-1^_3_(f_1_(x_1_)) + f^-1^_3_(f_2_(x_2_)) + x_3 _= f^-1^_3_(i)     [3]

f^-1^_3_(f_2_(f^-1^_2_(f_1_(x_1_)) + x_2_)) + x_3 _= f^-1^_3_(i)     [4]

f^-1^_3_(f_1_(f^-1^_1_(f_2_(x_2_)) + x_1_)) + x_3 _= f^-1^_3_(i)     [5]

f_2_(f^-1^_2_(f_1_(x_1_)) + x_2_) + f_3_(x_3_) = i     [6]

f_1_((f^-1^_1_(f_2_(x_2_)) + x_1_) + f_3_(x_3_) = i     [7]

f_3 _^-1^(f_1_(x_1_) + f_2_(x_2_)) + x_3 _= f^-1^_3_(i)     [8]

f_3_(f^-1^_3_(f_1_(x_1_)) + f^-1^_3_(f_2_(x_2_))) + f_3_(x_3_) = i     [9]

Most of these equations use a mixed definition for "additivity" namely dose (or Loewe) and response additivity as mentioned above.

Cyclic permutation of these seven equations leads to the other 14 equations.

By further investigation it can be shown that these overall 22 equations are complete which would be beyond the scope of this paper.

For practical use it will be sufficient to examine the two outer surfaces which contain the "volume of additivity" and to determine where the "point of therapy" is located. As shown later, it is not evident what these two surfaces are.

### Example

After considering the general purpose of isobologram analysis, the following attempt is used to demonstrate a special example:

f_j _(x_j_) = a_j _ln x_j _+ b_j_, j ∈ {1, 2}, f_3 _(x_3_) = a_3 _x_3 _+ b_3 _    [10]

In this notation f_3 _denotes the effect of (X)RT and a_j_, b_j _are parameters to be determined from the experiment. The "effect" may be "cell death induction" or in our case "induction of apoptosis". All coefficients and required parameters are listed in Tab. 1 and Tab. 2.

It depends on the experimental design whether [10] is useful or not. For clonogenic assays in vitro or growth delay experiments in vivo one should actually use linear-quadratic approaches.

One must keep in mind that the used equations describe extrapolated curves which try to predict the effect for doses which cannot be tested (so it is more reliable to use an established model).

Now one has to evaluate all calculated equations. The result is a set of 22 implicitly given surfaces which can be plotted.

With respect to [10] one has to remark that some of the equations are identical (as f_3 _is linear).

The corresponding solutions in an explicit manner will be of the form x_k _= g(x_i_, x_j_). Beside the three-dimensional representation it will be useful to plot the cuts through the point (d_1_|d_2_|d_3_). This is easily performed by setting x_i _= d_i_, i ∈ {1, 2, 3}.

Here are some of the solutions (the number of the formula with a star corresponds to the above mentioned equation without star):

x3=1a3(−b1−b2−b3+i−a1ln⁡x1−a2ln⁡x2)     [2∗]
 MathType@MTEF@5@5@+=feaafiart1ev1aaatCvAUfKttLearuWrP9MDH5MBPbIqV92AaeXatLxBI9gBaebbnrfifHhDYfgasaacH8akY=wiFfYdH8Gipec8Eeeu0xXdbba9frFj0=OqFfea0dXdd9vqai=hGuQ8kuc9pgc9s8qqaq=dirpe0xb9q8qiLsFr0=vr0=vr0dc8meaabaqaciaacaGaaeqabaqabeGadaaakeaacqqG4baEdaWgaaWcbaGaeG4mamdabeaakiabg2da9maalaaabaGaeGymaedabaGaeeyyae2aaSbaaSqaaiabiodaZaqabaaaaOWaaeWaaeaacqGHsislcqqGIbGydaWgaaWcbaGaeGymaedabeaakiabgkHiTiabbkgaInaaBaaaleaacqaIYaGmaeqaaOGaeyOeI0IaeeOyai2aaSbaaSqaaiabiodaZaqabaGccqGHRaWkcqqGPbqAcqGHsislcqqGHbqydaWgaaWcbaGaeGymaedabeaakiGbcYgaSjabc6gaUjabbIha4naaBaaaleaacqaIXaqmaeqaaOGaeyOeI0Iaeeyyae2aaSbaaSqaaiabikdaYaqabaGccyGGSbaBcqGGUbGBcqqG4baEdaWgaaWcbaGaeGOmaidabeaaaOGaayjkaiaawMcaaiaaxMaacaWLjaGaei4waSLaeGOmaiZaaWbaaSqabeaacqGHxiIkaaGccqGGDbqxaaa@5908@

x3=1a3(−b1−b2+b3+i−a1ln⁡x1−a2ln⁡x2)     [3∗]
 MathType@MTEF@5@5@+=feaafiart1ev1aaatCvAUfKttLearuWrP9MDH5MBPbIqV92AaeXatLxBI9gBaebbnrfifHhDYfgasaacH8akY=wiFfYdH8Gipec8Eeeu0xXdbba9frFj0=OqFfea0dXdd9vqai=hGuQ8kuc9pgc9s8qqaq=dirpe0xb9q8qiLsFr0=vr0=vr0dc8meaabaqaciaacaGaaeqabaqabeGadaaakeaacqqG4baEdaWgaaWcbaGaeG4mamdabeaakiabg2da9maalaaabaGaeGymaedabaGaeeyyae2aaSbaaSqaaiabiodaZaqabaaaaOWaaeWaaeaacqGHsislcqqGIbGydaWgaaWcbaGaeGymaedabeaakiabgkHiTiabbkgaInaaBaaaleaacqaIYaGmaeqaaOGaey4kaSIaeeOyai2aaSbaaSqaaiabiodaZaqabaGccqGHRaWkcqqGPbqAcqGHsislcqqGHbqydaWgaaWcbaGaeGymaedabeaakiGbcYgaSjabc6gaUjabbIha4naaBaaaleaacqaIXaqmaeqaaOGaeyOeI0Iaeeyyae2aaSbaaSqaaiabikdaYaqabaGccyGGSbaBcqGGUbGBcqqG4baEdaWgaaWcbaGaeGOmaidabeaaaOGaayjkaiaawMcaaiaaxMaacaWLjaGaei4waSLaeG4mamZaaWbaaSqabeaacqGHxiIkaaGccqGGDbqxaaa@58FF@

x3=1a3(i−b2−a2ln⁡(e1a2(a1ln⁡x1+b1−b2)+x2))     [4∗]
 MathType@MTEF@5@5@+=feaafiart1ev1aaatCvAUfKttLearuWrP9MDH5MBPbIqV92AaeXatLxBI9gBaebbnrfifHhDYfgasaacH8akY=wiFfYdH8Gipec8Eeeu0xXdbba9frFj0=OqFfea0dXdd9vqai=hGuQ8kuc9pgc9s8qqaq=dirpe0xb9q8qiLsFr0=vr0=vr0dc8meaabaqaciaacaGaaeqabaqabeGadaaakeaacqqG4baEdaWgaaWcbaGaeG4mamdabeaakiabg2da9maalaaabaGaeGymaedabaGaeeyyae2aaSbaaSqaaiabiodaZaqabaaaaOWaaeWaaeaacqqGPbqAcqGHsislcqqGIbGydaWgaaWcbaGaeGOmaidabeaakiabgkHiTiabbggaHnaaBaaaleaacqaIYaGmaeqaaOGagiiBaWMaeiOBa42aaeWaaeaacqqGLbqzdaahaaWcbeqaamaalaaabaGaeGymaedabaGaeeyyae2aaSbaaWqaaiabikdaYaqabaaaaSWaaeWaaeaacqqGHbqydaWgaaadbaGaeGymaedabeaaliGbcYgaSjabc6gaUjabbIha4naaBaaameaacqaIXaqmaeqaaSGaey4kaSIaeeOyai2aaSbaaWqaaiabigdaXaqabaWccqGHsislcqqGIbGydaWgaaadbaGaeGOmaidabeaaaSGaayjkaiaawMcaaaaakiabgUcaRiabbIha4naaBaaaleaacqaIYaGmaeqaaaGccaGLOaGaayzkaaaacaGLOaGaayzkaaGaaCzcaiaaxMaacqGGBbWwcqaI0aandaahaaWcbeqaaiabgEHiQaaakiabc2faDbaa@6027@

x3=1a3(i−b1−a1ln⁡(e1a1(a2ln⁡x2+b2−b1)+x1))     [5∗]
 MathType@MTEF@5@5@+=feaafiart1ev1aaatCvAUfKttLearuWrP9MDH5MBPbIqV92AaeXatLxBI9gBamXvP5wqSXMqHnxAJn0BKvguHDwzZbqegyvzYrwyUfgarqqtubsr4rNCHbGeaGqiA8vkIkVAFgIELiFeLkFeLk=iY=Hhbbf9v8qqaqFr0xc9pk0xbba9q8WqFfeaY=biLkVcLq=JHqVepeea0=as0db9vqpepesP0xe9Fve9Fve9GapdbaqaaeGacaGaaiaabeqaamqadiabaaGcbaGaeeiEaG3aaSbaaSqaaiabiodaZaqabaGccqGH9aqpdaWcaaqaaiabigdaXaqaaiabbggaHnaaBaaaleaacqaIZaWmaeqaaaaakmaabmaabaGaeeyAaKMaeyOeI0IaeeOyai2aaSbaaSqaaiabigdaXaqabaGccqGHsislcqqGHbqydaWgaaWcbaGaeGymaedabeaakiGbcYgaSjabc6gaUnaabmaabaGaeeyzau2aaWbaaSqabeaadaWcaaqaaiabigdaXaqaaiabbggaHnaaBaaameaacqaIXaqmaeqaaaaalmaabmaabaGaeeyyae2aaSbaaWqaaiabikdaYaqabaWccyGGSbaBcqGGUbGBcqqG4baEdaWgaaadbaGaeGOmaidabeaaliabgUcaRiabbkgaInaaBaaameaacqaIYaGmaeqaaSGaeyOeI0IaeeOyai2aaSbaaWqaaiabigdaXaqabaaaliaawIcacaGLPaaaaaGccqGHRaWkcqqG4baEdaWgaaWcbaGaeGymaedabeaaaOGaayjkaiaawMcaaaGaayjkaiaawMcaaiaaxMaacaWLjaGaei4waSLaeGynauZaaWbaaSqabeaacqGHxiIkaaGccqGGDbqxaaa@7064@

x3=1a3(i−b2−b3−a2ln⁡(e1a2(a1ln⁡x1+b1−b2)+x2))     [6∗]
 MathType@MTEF@5@5@+=feaafiart1ev1aaatCvAUfKttLearuWrP9MDH5MBPbIqV92AaeXatLxBI9gBaebbnrfifHhDYfgasaacH8akY=wiFfYdH8Gipec8Eeeu0xXdbba9frFj0=OqFfea0dXdd9vqai=hGuQ8kuc9pgc9s8qqaq=dirpe0xb9q8qiLsFr0=vr0=vr0dc8meaabaqaciaacaGaaeqabaqabeGadaaakeaacqqG4baEdaWgaaWcbaGaeG4mamdabeaakiabg2da9maalaaabaGaeGymaedabaGaeeyyae2aaSbaaSqaaiabiodaZaqabaaaaOWaaeWaaeaacqqGPbqAcqGHsislcqqGIbGydaWgaaWcbaGaeGOmaidabeaakiabgkHiTiabbkgaInaaBaaaleaacqaIZaWmaeqaaOGaeyOeI0Iaeeyyae2aaSbaaSqaaiabikdaYaqabaGccyGGSbaBcqGGUbGBdaqadaqaaiabbwgaLnaaCaaaleqabaWaaSaaaeaacqaIXaqmaeaacqqGHbqydaWgaaadbaGaeGOmaidabeaaaaWcdaqadaqaaiabbggaHnaaBaaameaacqaIXaqmaeqaaSGagiiBaWMaeiOBa4MaeeiEaG3aaSbaaWqaaiabigdaXaqabaWccqGHRaWkcqqGIbGydaWgaaadbaGaeGymaedabeaaliabgkHiTiabbkgaInaaBaaameaacqaIYaGmaeqaaaWccaGLOaGaayzkaaaaaOGaey4kaSIaeeiEaG3aaSbaaSqaaiabikdaYaqabaaakiaawIcacaGLPaaaaiaawIcacaGLPaaacaWLjaGaaCzcaiabcUfaBjabiAda2maaCaaaleqabaGaey4fIOcaaOGaeiyxa0faaa@638D@

x3=1a3(i−b1−b3−a1ln⁡(e1a1(a2ln⁡x2+b2−b1)+x1))     [7∗]
 MathType@MTEF@5@5@+=feaafiart1ev1aaatCvAUfKttLearuWrP9MDH5MBPbIqV92AaeXatLxBI9gBaebbnrfifHhDYfgasaacH8akY=wiFfYdH8Gipec8Eeeu0xXdbba9frFj0=OqFfea0dXdd9vqai=hGuQ8kuc9pgc9s8qqaq=dirpe0xb9q8qiLsFr0=vr0=vr0dc8meaabaqaciaacaGaaeqabaqabeGadaaakeaacqqG4baEdaWgaaWcbaGaeG4mamdabeaakiabg2da9maalaaabaGaeGymaedabaGaeeyyae2aaSbaaSqaaiabiodaZaqabaaaaOWaaeWaaeaacqqGPbqAcqGHsislcqqGIbGydaWgaaWcbaGaeGymaedabeaakiabgkHiTiabbkgaInaaBaaaleaacqaIZaWmaeqaaOGaeyOeI0Iaeeyyae2aaSbaaSqaaiabigdaXaqabaGccyGGSbaBcqGGUbGBdaqadaqaaiabbwgaLnaaCaaaleqabaWaaSaaaeaacqaIXaqmaeaacqqGHbqydaWgaaadbaGaeGymaedabeaaaaWcdaqadaqaaiabbggaHnaaBaaameaacqaIYaGmaeqaaSGagiiBaWMaeiOBa4MaeeiEaG3aaSbaaWqaaiabikdaYaqabaWccqGHRaWkcqqGIbGydaWgaaadbaGaeGOmaidabeaaliabgkHiTiabbkgaInaaBaaameaacqaIXaqmaeqaaaWccaGLOaGaayzkaaaaaOGaey4kaSIaeeiEaG3aaSbaaSqaaiabigdaXaqabaaakiaawIcacaGLPaaaaiaawIcacaGLPaaacaWLjaGaaCzcaiabcUfaBjabiEda3maaCaaaleqabaGaey4fIOcaaOGaeiyxa0faaa@638B@

x3=1a3(i−b1−b2−a1ln⁡x1−a2ln⁡x2)     [8∗]
 MathType@MTEF@5@5@+=feaafiart1ev1aaatCvAUfKttLearuWrP9MDH5MBPbIqV92AaeXatLxBI9gBaebbnrfifHhDYfgasaacH8akY=wiFfYdH8Gipec8Eeeu0xXdbba9frFj0=OqFfea0dXdd9vqai=hGuQ8kuc9pgc9s8qqaq=dirpe0xb9q8qiLsFr0=vr0=vr0dc8meaabaqaciaacaGaaeqabaqabeGadaaakeaacqqG4baEdaWgaaWcbaGaeG4mamdabeaakiabg2da9maalaaabaGaeGymaedabaGaeeyyae2aaSbaaSqaaiabiodaZaqabaaaaOWaaeWaaeaacqqGPbqAcqGHsislcqqGIbGydaWgaaWcbaGaeGymaedabeaakiabgkHiTiabbkgaInaaBaaaleaacqaIYaGmaeqaaOGaeyOeI0Iaeeyyae2aaSbaaSqaaiabigdaXaqabaGccyGGSbaBcqGGUbGBcqqG4baEdaWgaaWcbaGaeGymaedabeaakiabgkHiTiabbggaHnaaBaaaleaacqaIYaGmaeqaaOGagiiBaWMaeiOBa4MaeeiEaG3aaSbaaSqaaiabikdaYaqabaaakiaawIcacaGLPaaacaWLjaGaaCzcaiabcUfaBjabiIda4maaCaaaleqabaGaey4fIOcaaOGaeiyxa0faaa@54D0@

[8] and [9] have an identical solution because of the above mentioned linearity of f_3_. Overall, there are 5 equations which are redundant so that there are 17 distinct surfaces at the end.

Some of the surfaces have no real solutions in certain domains of the space. In these special cases a 2D representation helps to decide whether the point of therapy lies in the domain of synergism or not.

It can be shown that the solutions are unique (which is a consequence of the bijective attempt [10]). This proof would be beyond the scope of the paper.

**Figure 2:**
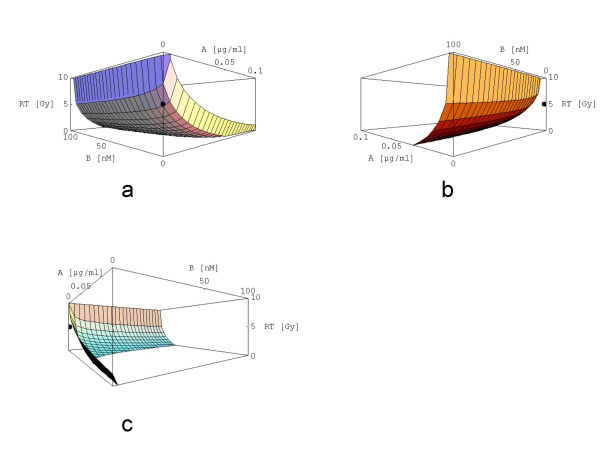
a, b, c: Shown is the surface of strict response additivity for the triple therapy 5 [Gy] + 0.001 [μg/ml] A + 1 [nM] B from three different perspectives. The axes are labeled with the dose/concentrations of the single agents (x_1_: A, x_2_: B, x_3_: XRT) in a right-handed cartesian coordinate system. The plot ranges are [0, 10] for XRT, [0, 0.1] for A and [0, 100] for B. The black dot marks the "point of therapy" namely (0.001|1|5); x_1 _= 0.001 and x_2 _= 1 means that the point is close to the x_3_-axis. The "point of therapy" lies in the middle of the plot range [0, 10].

**Figure 3:**
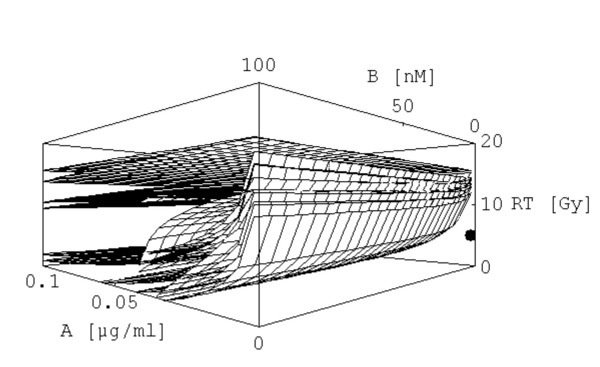
All 17 surfaces are plotted for the therapy (0.001|1|5), the point of therapy was also included. In order to show all these surfaces the x_3_-axis (RT) was extended.

The solution of [2] is shown in Fig. 2a – c, the plot is presented from three different perspectives. All 22 equations are plotted together in Fig. 3. By analysing the surfaces more accurately it seems that [2] represents the innermost surface.

**Figure 4:**
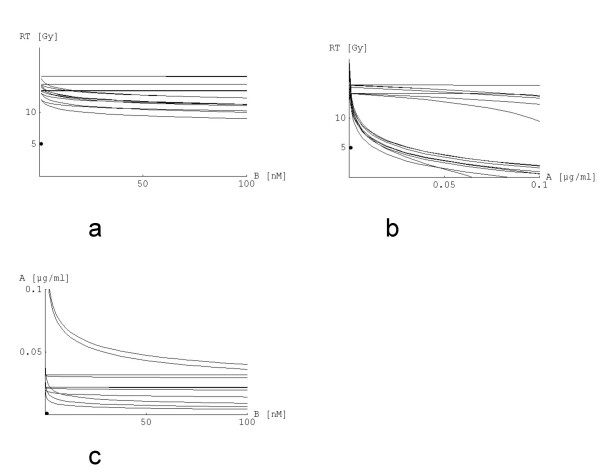
a - c: Displayed are the 2D cuts through the three-dimensional plot in Fig. 3; shown are the layers which contain the point of therapy (0.001|1|5). Fig. 4 a: x_2 _- x_3_-plane (x_1 _= d_1 _is fixed), b: x_1 _- x_3_-plane, c: x_2 _- x_1_-plane.

As it is a question of perspective whether one realizes that the point lies under the innermost surface, one can plot a two-dimensional graph as indicated before which is done in Fig. 4. From Fig. 4b it can be deduced that [2] is not completely the innermost surface.

It is interesting that in Fig. 4 there seem to be less curves than equations. There are two simple reasons: 1) some equations are identical as mentioned above, 2) some equations produce different results but are very similar to other equations so that they cannot be differentiated visually.

**Figure 5:**
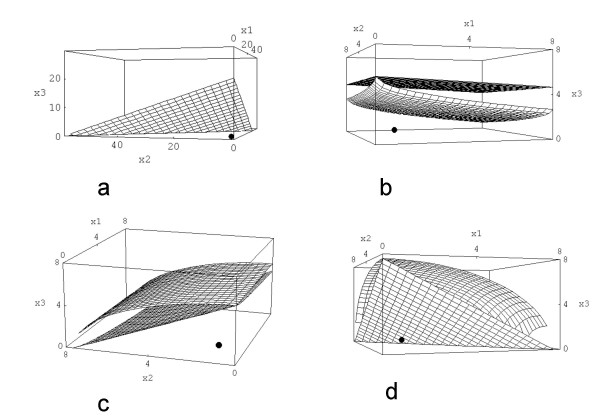
a - d: This shows the volume of additivity for four different cases; displayed are only inner- and outermost surface of additivity, the point of therapy is again included. 5 a shows the case for three linear dose-response-relationships. As expected one gets a single plane (corresponding to the straight line in the 2D case). 5 b is calculated by using one linear and two linear-quadratic equations, 5 c uses two linear and one linear-quadratic equation and 5 d is calculated by three linear-quadratic equations.

The isobole surfaces for linear-quadratic approaches were also determined according to the above mentioned equations. We plotted inner- and outermost surface as well as the point of therapy. The results are shown in Fig. 5a – d.

We provide (as a supplement to this paper) two small programmes which the reader can use for his own experimental data (a detailed description is also included) [see Additional files 1, 2 and 3].

### Statistics

Similar as for two-dimensional isobolograms [9] a separate statistical analysis is required in our case. As one assumes the regression curves for the agents/XRT to be "exact", it is possible to calculate a new isobologram which corresponds to the lower limit of the 95 % confidence interval of the isodose (which corresponds to [i - 1,96 × SEM]). So we are able to elucidate if the triple remains synergistic. In this case we would be allowed to call the synergism "significant".

## Discussion

Starting from the semantic definition of "synergy" and the problem to evaluate a combination of three modalities we developed a system of 22 equations which enable the user to derive the correct type of interaction.

This is a new aspect according to the classically used "combination index" [10] which only focuses on one of the 22 surfaces.

From the reported theory the following may be obtained:

1) It leads to a far wider concept of additivity. Thus it is important to define what definition is used.

2) It is often difficult to get out the mechanisms on the molecular layer but isobologram analysis has managed to handle these combined therapies and allows to deduce some of the biological effects behind the therapy.

This means that a synergism can under certain circumstances be a useful tool to elicit which combinations might make sense for clinical trials.

For this purpose strict conditions should be set up to determine which one of the combinations is really promising. The given equations (in their general form) provide the necessary mathematical formalism for this purpose.

One problem remains: how can we decide whether a given triple therapy acts in a "more synergistic" way than a combination of just two modalities?

It was only shown that the triple therapy is synergistic with respect to the effects of the single modalities. When using isobologram analysis there is no other possibility as a double combination is not feasible as a free parameter.

**Figure 6:**
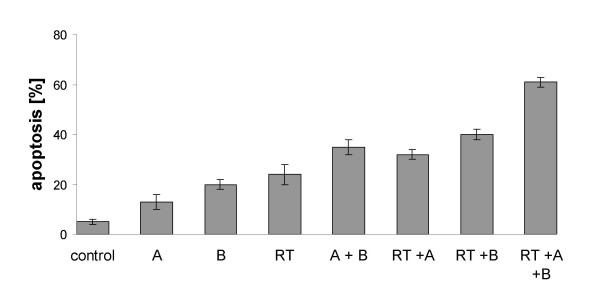
This bar graph displays control, single, double and triple therapies for the therapy (0.001|1|5). It is important that RT + A + B is significantly better than the corresponding double combinations A + B, RT + A and RT + B. Synergy alone is not sufficient to guarantee this.

For practical purposes it is necessary to determine whether a given triple therapy is a "good" therapy. Our attempt is to elucidate if the combination is synergistic and as a second criterion it has to be significantly better than the corresponding double combinations (which means that synergy is a necessary, but no sufficient criterion for a "good" therapy); one additionally has to compare the effects of double therapies and triple therapy in a bar graph. This is shown in Fig. 6.

## Materials and methods

Mathematical calculations and graphical evaluation were performed by the use of Mathematica 5.2 for students^®^, Wolfram Research (Friedrichsdorf, Germany).

**Table 1:** Coefficients for the parametric representations of the dose-response-relationships

***a***_**1**_	***a***_**2**_	***a***_**3**_	***b***_**1**_	***b***_**2**_	***b***_**3**_
8.5	2.0	3.1	65.6	4.3	3.1

**Table 2:** Doses for the triple therapy RT + A + B and level of isodose

***i [%]***	***d***_**1 **_***[μg/ml]***	***d***_**2**_***[nM]***	***d***_**3**_***[Gy]***
52	0.001	1	5

## Supplementary Material

Additional File 1 Isobolyzer - a tool for isobologram analysis of triple therapies. A help-file for the two delivered programmes, containing advice for installation, use and handling. Click here for file

Additional File 2 Isobolyzer for linear-quadratic dose-response-relationships. This Excel macro enables the user to type in his own experimental data. The used dose-response-relationships are linear-quadratic. Click here for file

Additional File 3 Isobolyzer for two logarithmic and one linear dose-response-relationship. This Excel macro enables the user to type in his own experimental data. The used dose-response-relationships are linear and logarithmic. Click here for file
