# Supplementary material for: Isobologram analysis of triple therapies
Source: Radiat Oncol. 2006 Oct 17;1:39. doi: 10.1186/1748-717X-1-39 (PMC1634864; doi:10.1186/1748-717X-1-39)
Supplement: Additional File 1 — Isobolyzer - a tool for isobologram analysis of triple therapies. A help-file for the two delivered programmes, containing advice for installation, use and handling. [file 1748-717X-1-39-S1.pdf]

Isobolyzer – a tool for isobologram analysis of triple therapies

Attention: Although we carefully tried to optimize the programme, we are not able to adopt any responsibilities for results that were obtained with it.

Overview and purpose

Isobologram analysis is useful to determine if several agents act in a synergistic way. This tool calculates according to our online paper 2D cuts through the 3D surfaces. A point of therapy below the curves in all three diagrams indicates synergy.

As it is complicated to plot 3D surfaces without using special software, e. g. Mathematica®, we decided to provide a tool based on Microsoft® Excel 2000 which calculates and plots these 2D cuts. From these diagrams one can deduce whether there is an underlying synergism or not.

Prerequisites

It is necessary to use Microsoft® Excel 2000 or higher. Macros should be allowed; otherwise the programme cannot be started.

Note: We used the German decimal system.

First steps

You should save the Excel-files and the help file in the same directory (you can invoke the help file directly or from the macro within the Excel-files). The Excel-files contain different dose-response-relationships: “Isobolyzer_linq.xls” handles three linear-quadratic dose-response-relationships (which can also be used as pure linear equations) and “Isobolyzer_logl.xls” contains two logarithmic and one linear dose-response-relationship (log-linear).

General notes

Yellow marked cells contain values that you should change for your calculation. We supply the programme with default values which you can use for the first time to see how the programme works. Of course you should change these values to match your own demands.

Important:

- Clear the sheets before you start a new calculation
- Do not change any parameter before you clear the sheets
- Confirm your input in a cell with carriage return (otherwise you cannot use the push buttons)

Explanation of the different sheets

Push buttons

On every sheet there are three push buttons which are identical and enable you to use the programme in every sheet of the Excel workbook. After pushing a button it will take some time to execute the command. During this time you will see an hour glass and any action is not possible. Please wait until the hour glass disappears.

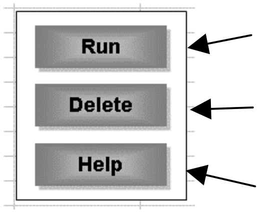

Starts the calculation in all sheets. Clear first if calculated values exist (see notes above)

Clear all sheets. All calculated values will be deleted (see notes above)

Starts, if necessary, the pdf-reader and shows the help-file (to see this file you must have a pdf-reader from Adobe®)

First sheet (Variables)

Within this sheet you're able to specify the dose-response-relationships of the single agents (dependent on the file).

	A	B	C	D	E	F	G	H
1	parameter						definitions	
2	a1	8,5	b1	65,6			Therapy No. 1	y1 = a1 lnx1 + b1
3	a2	2	b2	4,3			Therapy No. 2	y2 = a2 lnx2 + b2
4	a3	3,1	b3	3,1			Therapy No. 3	y3 = a3 x3 + b3
5	d1	0,001					d1	dose of therapy 1
6	d2	1					d2	dose of therapy 2
7	d3	5					d3	dose of therapy 3
8	i	52					i	effect of combination (isodose)

In "Isobolyzer_logl.xls" the parameters a1 to a3 and b1 to b3 are the coefficients of the used equations (see figure). Further information on these equations can be found in our online publication "Isobologram analysis of triple therapies", Radiation Oncology. "Isobolyzer_linq.xls" contains the parameters a1 to a3, b1 to b3 and c1 to c3 for the linear-quadratic equations. We tried to cover a broad range of problems but if other equations have to be implemented, please let us know.

It is important to remark that the parameter "i" is the isodose effect which means that the amount of killed cells, apoptosis or whatever should be used and not the pure survival!

Sheets for Calculation

The yellow marked cells contain the values of the considered dose interval (you should use minimum and maximum dose values). The increment is equivalent to the step size which is used to plot the diagram.

<table>
<tr><th></th><th>A</th><th>B</th><th>C</th></tr>
<tr><td>1</td><td>increment</td><td colspan="2">interval</td></tr>
<tr><td>2</td><td></td><td>start</td><td>end</td></tr>
<tr><td>3</td><td>0,001</td><td>0,001</td><td>0,1</td></tr>
<tr><td>4</td><td></td><td></td><td></td></tr>
<tr><td>5</td><td>(d1|d2)</td><td>0,001</td><td>1</td></tr>
</table>

x2 – x3 sheet

This sheet sets x1=d1 (which is the used dose of therapy 1 within the triple therapy) and calculates the 2D cut through the 3D surface and displays the point (d2|d3).

x1 – x3 and x1 – x2 sheets

These sheets are defined analogously as x2 – x3 (you also have to set increment an interval borders as previously described).

Possible problems

When choosing the interval scale, it may happen that curves are plotted in a range that does not make sense (as Excel automatically scales the y-axis; the x-axis is chosen before the calculation). In this case you should manually choose the correct range, i. e. take the range which describes the dose range better. This example is included in "Isobolyzer_logl.xls", sheet "x1 – x2". The range which was automatically generated is very large; so one has to choose the dose range [1, 100] and the diagram becomes more understandable.

Sometimes there are cells which are left free. Then there's possibly no solution for a given value which may occur when complex solutions are regarded. Sometimes there occur negative values which do not correspond to a biological solution. These should be omitted from the diagram (which is certainly fulfilled if you choose the range properly). This is related to the used equations. Here one should

mention that these equations are (for some problems) not suitable at the origin or for higer doses (as they do not contain a saturation factor). This is in part corrected by conditions within the functions (they cannot produce negative values or do not accept a negative parameter).

One important point is the step size or interval increment. If one sets it too high, the curves are perhaps not entirely plotted. But if it is too low the calculations are probably very time intensive. One has to find a good compromise between these two possibilities.

Evaluation

We're interested that you have no trouble with using this programme. Anyhow let us know if there are any problems or if you think there is a mistake within the results (this can never be completely excluded). We try to answer your questions as soon as possible but please keep in mind that it will possibly take some time.

Acknowledgement

We cordially thank I. Niyazi for helpful advice with VBA-programming.

Correspondence

The mailing address of the corresponding author is maxi@niyazi.de.
